# Endoscopic ultrasound-guided hepaticogastrostomy without tract dilation using a novel ultra-tapered slim-delivery metallic stent

**DOI:** 10.1055/a-2552-0373

**Published:** 2025-03-20

**Authors:** Ritsuko Oishi, Haruo Miwa, Kazuki Endo, Hiromi Tsuchiya, Yuichi Suzuki, Kazushi Numata, Shin Maeda

**Affiliations:** 1 26437 Gastroenterological Center, Yokohama City University Medical Center, Yokohama, Japan; 2 Department of Gastroenterology, Yokohama City University Graduate School of Medicine, Yokohama, Japan

Endoscopic ultrasound-guided hepaticogastrostomy (EUS-HGS) has a high technical success rate; however, severe adverse events such as biliary peritonitis and stent migration may occur 1 . To reduce these risks, self-expandable metallic stent (SEMS) placement without tract dilation has been reported 2 3 . However, the fully-covered and small-diameter design of the SEMS carries a risk of migration 4 .

A novel SEMS with a 7-Fr slim delivery system (Niti-S EUS-BD system End Bare Single Flare, 8 mm × 12 cm; Taewoong Medical Co., Ltd., Gimpo, South Korea) features a cross-wire structure that maintains stiffness with a reduced delivery system diameter. It also has an ultra-tapered 0.9-mm tip that minimizes the gap with a 0.025-inch-diameter guidewire, which facilitates smooth insertion of the stent delivery system. Additionally, a partially covered proximal end and flared distal end can prevent stent migration ( Fig. 1 ). Herein, we report EUS-HGS without tract dilation using the novel SEMS ( Video 1 ).

**Fig. 1:**
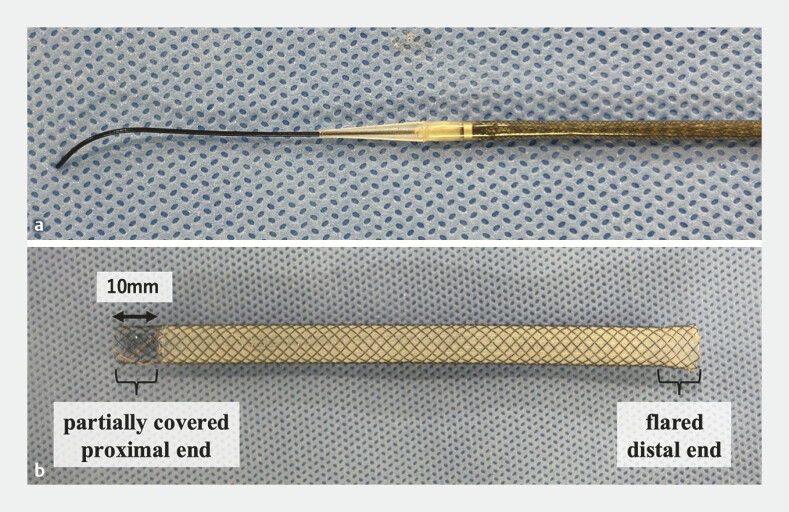
**a** A novel self-expandable metallic stent with a 7-Fr slim delivery system features an ultra-tapered 0.9-mm tip that minimizes the gap with a 0.025-inch-diameter guidewire. **b** The stent (8 mm × 12 cm) is structured by a cross-wire, partially covered proximal end and a flared distal end.

A novel ultra-tapered slim-delivery metallic stent was successfully placed without tract dilation during endoscopic ultrasound-guided hepaticogastrostomy while minimizing the risk of stent migration.Video 1

An 81-year-old woman with biliary obstruction caused by pancreatic head cancer initially underwent transpapillary SEMS placement. Three months later, EUS-HGS was planned for recurrent biliary obstruction due to complete stent migration ( Fig. 2 ). First, intrahepatic bile duct (B2) was punctured with a 19-gauge needle, and a 0.025-inch guidewire (VisiGlide 2; Olympus Medical Systems, Tokyo, Japan) was advanced into the common bile duct after contrast injection. Subsequently, the SEMS with a 7-Fr delivery system was inserted without tract dilation. The ultra-tapered tip passed smoothly into the bile duct, and the SEMS (8 mm × 12 cm) was successfully placed within 10 minutes ( Fig. 3 ). The patient showed improvement in jaundice and was discharged without adverse events.

**Fig. 2:**
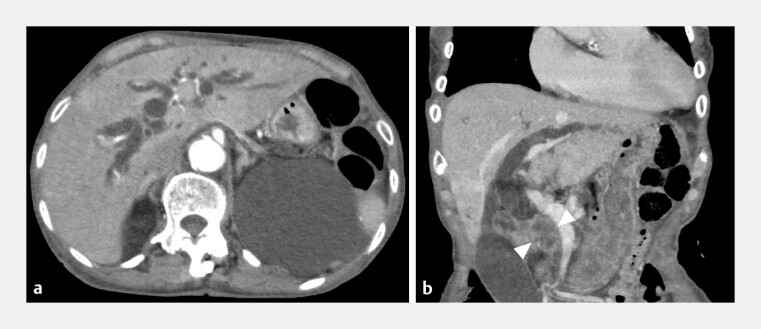
Computed tomography images show pancreatic head cancer (arrowheads) and dilated intrahepatic bile duct. **a** Axial plane. **b** Coronal plane.

**Fig. 3:**
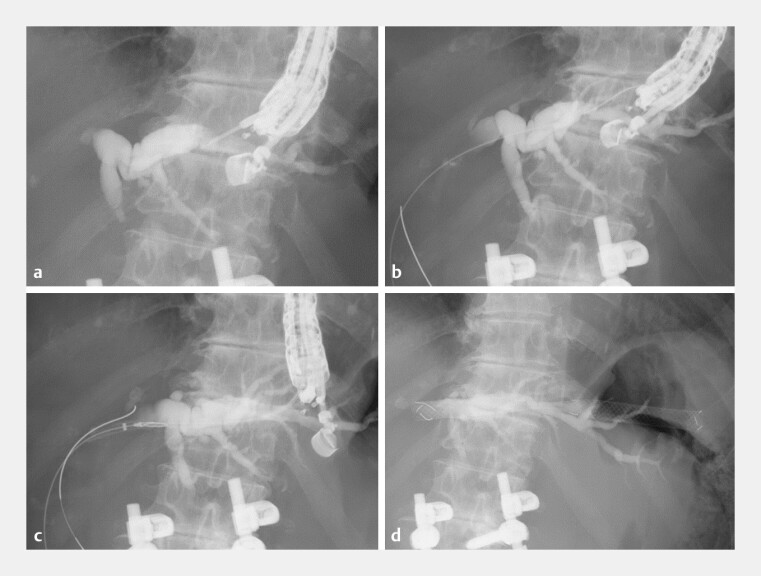
Endoscopic ultrasound-guided hepaticogastrostomy. **a** Intrahepatic bile duct (B2) is punctured with a 19-gauge needle. **b** A 0.025-inch guidewire is advanced into the common bile duct. **c** A 7-Fr delivery system of the stent is inserted without tract dilation. **d** The self-expandable metallic stent (8 mm × 12 cm) is successfully placed within 10 minutes.

To the best of our knowledge, this is the first case of EUS-HGS without tract dilation using the novel SEMS with an ultra-tapered slim delivery system. This simple technique can offer a safer approach for EUS-HGS.

Endoscopy_UCTN_Code_TTT_1AS_2AH

## Notes

Endoscopy E-Videos https://eref.thieme.de/e-videos
*E-Videos* is an open access online section of the journal *Endoscopy* , reporting on interesting cases and new techniques in gastroenterological endoscopy. All papers include a high-quality video and are published with a Creative Commons CC-BY license. Endoscopy E-Videos qualify for HINARI discounts and waivers and eligibility is automatically checked during the submission process. We grant 100% waivers to articles whose corresponding authors are based in Group A countries and 50% waivers to those who are based in Group B countries as classified by Research4Life (see: https://www.research4life.org/access/eligibility/ ). This section has its own submission website at https://mc.manuscriptcentral.com/e-videos .
